# 1-[(2-Methyl-8-quinol­yl)amino­methyl­ene]naphthalen-2(1*H*)-one

**DOI:** 10.1107/S1600536809048296

**Published:** 2009-11-21

**Authors:** Kentaro Takano, Masayuki Takahashi, Takaaki Fukushima, Takashi Shibahara

**Affiliations:** a Department of Chemistry, Okayama University of Science, Ridai-cho, Kita-ku, Okayama 700-0005, Japan

## Abstract

The mol­ecule of the title compound, C_21_H_16_N_2_O, exists in the keto form and the C=O and N—H bonds are mutually *cis* in the crystal structure, although an enol form would be possible through tautomerism. The dihedral angle between the quinoline and the naphthalene systems is 22.04 (2)°. A bifurcated intramolecular N—H⋯(O,N) hydrogen bond is present.

## Related literature

The title compound was prepared and the structure determined, to explore the substituent effects on the fluorescence of metal complexes of 2-hydr­oxy-1-naphthaldehydene-8-amino­quinoline (II), a fluorescent reagent for molybdenum (Jiang *et al.*, 2001 ▶) and beryllium (Jiang & He, 2003 ▶). For the structure of (II), see: Sakane *et al.* (2006 ▶). For the structures of its metal complexes with technetium, vanadium and tin, see: Tisato *et al.* (1990 ▶), Asgedom *et al.* (1996 ▶) and Takano & Shibahara (2008 ▶), respectively.
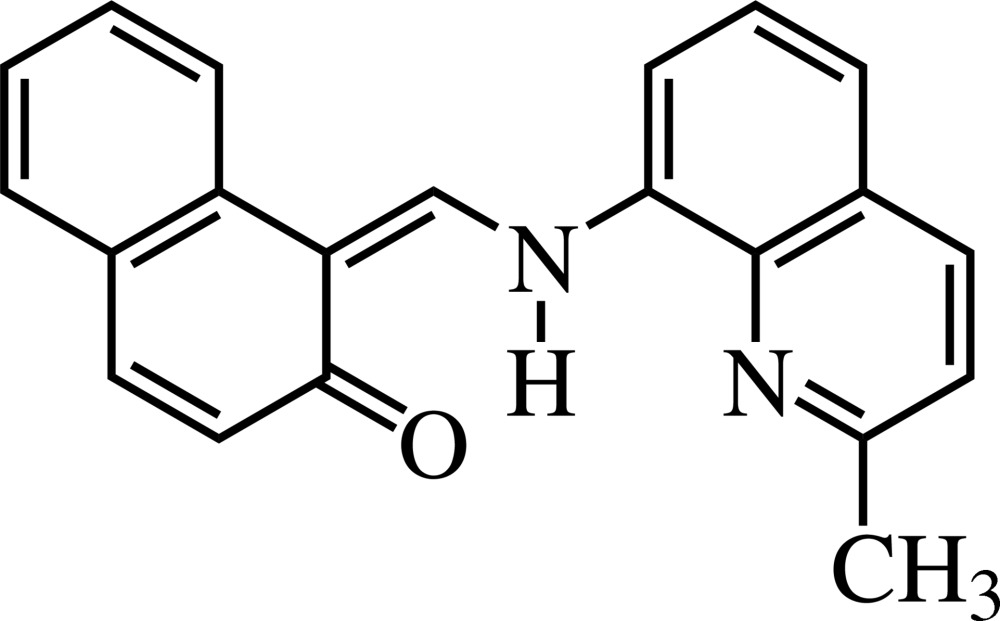


## Experimental

### 

#### Crystal data

C_21_H_16_N_2_O
*M* *_r_* = 312.37
Monoclinic, 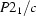

*a* = 10.6300 (19) Å
*b* = 15.0760 (17) Å
*c* = 10.9853 (17) Å
β = 117.111 (7)°
*V* = 1567.0 (4) Å^3^
*Z* = 4
Mo *K*α radiation
μ = 0.08 mm^−1^
*T* = 93 K
0.63 × 0.56 × 0.32 mm

#### Data collection

Rigaku Mercury diffractometer
Absorption correction: multi-scan (Jacobson, 1998 ▶) *T*_min_ = 0.950, *T*_max_ = 0.975
17775 measured reflections
4478 independent reflections
4145 reflections with *F* ^2^ > 2.0σ(*F* ^2^)
*R*_int_ = 0.038

#### Refinement

*R*[*F* ^2^ > 2σ(*F* ^2^)] = 0.046
*wR*(*F* ^2^) = 0.127
*S* = 1.01
4478 reflections
218 parameters
All H-atom parameters refined
Δρ_max_ = 0.49 e Å^−3^
Δρ_min_ = −0.24 e Å^−3^

### 

Data collection: *CrystalClear* (Rigaku, 1999 ▶); cell refinement: *CrystalClear* ▶); data reduction: *CrystalStructure* (Rigaku, 2007 ▶); program(s) used to solve structure: *SIR97* (Altomare *et al.*, 1999 ▶); program(s) used to refine structure: *SHELXL97* (Sheldrick, 2008 ▶); molecular graphics: *CrystalStructure*; software used to prepare material for publication: *CrystalStructure*.

## Supplementary Material

Crystal structure: contains datablocks global, I. DOI: 10.1107/S1600536809048296/zs2016sup1.cif

Structure factors: contains datablocks I. DOI: 10.1107/S1600536809048296/zs2016Isup2.hkl

Additional supplementary materials:  crystallographic information; 3D view; checkCIF report

**Fig. 1.**
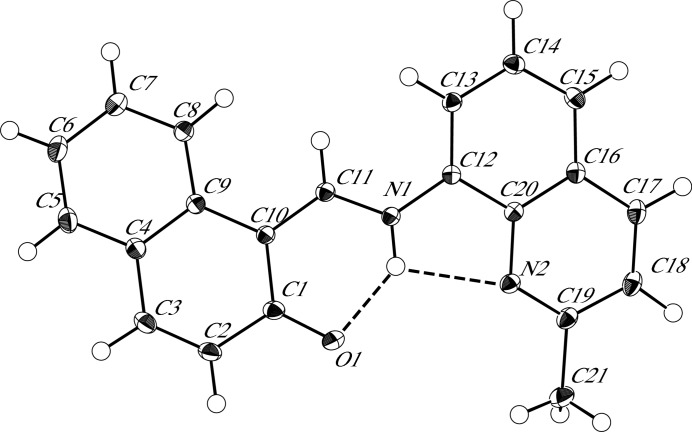
Molecular configuration and atom-numbering scheme for (I) with displacement ellipsoids drawn at the 50% probability level. Intramolecular hydrogen bonds are shown as dashed lines.

Enhanced figure: interactive version of Fig. 1

## Figures and Tables

**Table 1:** Hydrogen-bond geometry (Å, °)

*D*—H⋯*A*	*D*—H	H⋯*A*	*D*⋯*A*	*D*—H⋯*A*
N1—H8⋯O1	0.95	1.86	2.6094 (11)	133
N1—H8⋯N2	0.95	2.28	2.6714 (14)	104

**Fig. 2.**
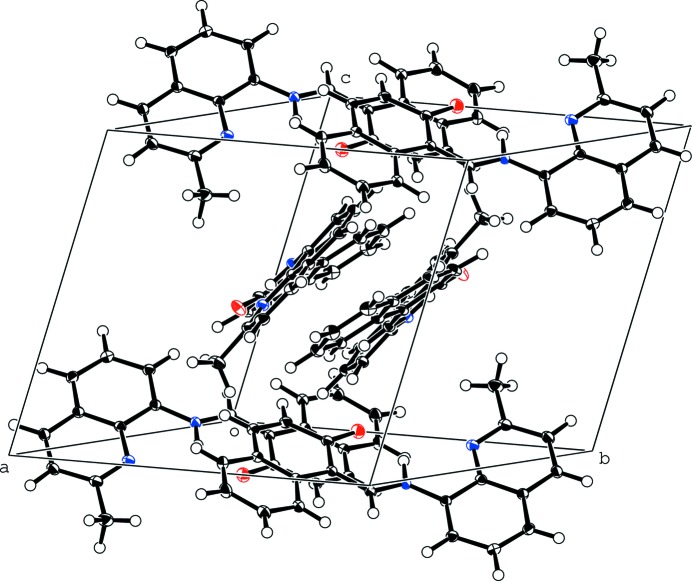
Molecular packing of (I) in the unit cell.

## supplementary crystallographic information

### Comment

In the present work, the title compound, 2-hydroxy-1-naphthaldehydene-8-amino-2-methylquinoline (C_21_H_16_N_2_O) (I) was prepared and the structure determined, to explore the substituent effects on the fluorescence of metal complexes of 2-hydroxy-1-naphthaldehydene-8-aminoquinoline (C_20_H_14_N_2_O) (II) (Sakane *et al.*, 2006). Compound (II) has been reported as a fluorescent reagent for molybdenum (Jiang *et al.*, 2001) and beryllium (Jiang & He, 2003). The X-ray structures of (II) (Sakane *et al.*, 2006) and its metal complexes with technetium (Tisato *et al.*, 1990), vanadium (Asgedom *et al.*, 1996), and tin (Takano & Shibahara, 2008) have been determined. The molecule of (I) (Fig. 1) exists in the keto form and the C═O and N—H bonds are mutually *cis* which is similar to that found in the structure of (II). In the structure of (I), N—H···O_carbonyl_ and N—H···N_pyridine_ intramolecular hydrogen bonds exist (Table 1) but there is no evidence of formal intermolecular hydrogen-bonding associations (Fig. 2).

### Experimental

A suspension of 8-amino-2-methylquinoline (158 mg, 1.0 mmol) and 2-hydroxy-1-naphthaldehyde (172 mg, mmol) in methanol (5 ml) was refluxed at 65°C for three hours. The resultant orange solution was ice-cooled to give an orange powder, which was washed with ice-cooled ethyl acetate: yield 262 mg. For recrystallization, the orange powder was dissolved in toluene (5 ml) at 60°C and kept overnight. Crystals were collected after cooling to room temperature. Yield 205 mg (66%). Anal. found: C, 80.53; H, 5.10; N, 8.95%. Calc. for C_21_H_16_N_2_O: C, 80.75; H, 5.16; N, 8.97%

### Refinement

The positions of all H atoms were located from difference maps and refined with restrained distances (N—H, 0.86 Å; C—H, 0.95 Å). The isotropic displacement parameters for these atoms were fixed at 1.2*U*_eq_ of their carrier atoms.

### Crystal data

**Table:**

C_21_H_16_N_2_O	*F*(000) = 656.00
*M**_r_* = 312.37	*D*_x_ = 1.324 Mg m^−^^3^
Monoclinic, *P*2_1_/*c*	Mo *K*α radiation, λ = 0.71070 Å
Hall symbol: -P 2ybc	Cell parameters from 4690 reflections
*a* = 10.6300 (19) Å	θ = 5.5–30.0°
*b* = 15.0760 (17) Å	µ = 0.08 mm^−^^1^
*c* = 10.9853 (17) Å	*T* = 93 K
β = 117.111 (7)°	Block, orange
*V* = 1567.0 (4) Å^3^	0.63 × 0.56 × 0.32 mm
*Z* = 4	

### Data collection

**Table:**

Rigaku Mercury diffractometer	4145 reflections with *F*^2^ > 2.0σ(*F*^2^)
Detector resolution: 7.31 pixels mm^-1^	*R*_int_ = 0.038
ω scans	θ_max_ = 30.0°
Absorption correction: multi-scan (Jacobson, 1998)	*h* = −14→14
*T*_min_ = 0.950, *T*_max_ = 0.975	*k* = −21→21
17775 measured reflections	*l* = −14→15
4478 independent reflections	

### Refinement

**Table:**

Refinement on *F*^2^	All H-atom parameters refined
*R*[*F*^2^ > 2σ(*F*^2^)] = 0.046	*w* = 1/[σ^2^(*F*_o_^2^) + (0.0732*P*)^2^ + 0.55*P*] where *P* = (*F*_o_^2^ + 2*F*_c_^2^)/3
*wR*(*F*^2^) = 0.127	(Δ/σ)_max_ < 0.001
*S* = 1.01	Δρ_max_ = 0.49 e Å^−^^3^
4478 reflections	Δρ_min_ = −0.24 e Å^−^^3^
218 parameters	

### Special details

**Table:**

Geometry. The dihedral angle between the quinoline (C11\simC19, N11) and the naphthalene (C21\simC30) rings is 22.04 (2)°: Mean deviations of the atoms from the former and latter planes are 0.0125 and 0.0186 Å, respectively. The corresponding dihedral angle of (II) is very small (1.67 (4)°).
Refinement. Refinement was performed using all reflections. The weighted *R*-factor (*wR*) and goodness of fit (*S*) are based on *F*^2^. *R*-factor (gt) are based on *F*. The threshold expression of *F*^2^ > 2.0 σ(*F*^2^) is used only for calculating *R*-factor (gt).

### Fractional atomic coordinates and isotropic or equivalent isotropic displacement parameters (Å^2^)

**Table:**

	*x*	*y*	*z*	*U*_iso_*/*U*_eq_	
O(1)	0.43362 (8)	0.17528 (5)	0.40947 (8)	0.02108 (16)	
N(1)	0.48710 (8)	0.31627 (5)	0.56290 (8)	0.01363 (15)	
N(2)	0.65907 (8)	0.36764 (5)	0.45922 (8)	0.01481 (16)	
C(20)	0.65577 (9)	0.42019 (6)	0.55895 (9)	0.01297 (16)	
C(13)	0.56130 (9)	0.44289 (6)	0.72100 (9)	0.01487 (17)	
C(11)	0.37459 (9)	0.29276 (6)	0.57781 (9)	0.01363 (17)	
C(9)	0.15828 (9)	0.20542 (6)	0.52257 (9)	0.01363 (17)	
C(12)	0.56770 (9)	0.39321 (6)	0.61849 (9)	0.01290 (16)	
C(10)	0.28825 (10)	0.22057 (6)	0.51161 (9)	0.01334 (16)	
C(19)	0.73821 (10)	0.39284 (7)	0.40156 (10)	0.01668 (18)	
C(16)	0.73327 (9)	0.50023 (6)	0.60430 (9)	0.01425 (17)	
C(17)	0.81977 (10)	0.52425 (6)	0.54213 (10)	0.01710 (18)	
C(1)	0.32746 (10)	0.16209 (6)	0.42995 (9)	0.01589 (17)	
C(18)	0.82189 (10)	0.47128 (7)	0.44228 (10)	0.0181 (2)	
C(15)	0.72083 (10)	0.55231 (6)	0.70543 (9)	0.01620 (18)	
C(4)	0.07181 (10)	0.13199 (6)	0.45402 (9)	0.01595 (18)	
C(8)	0.11163 (10)	0.26091 (6)	0.59766 (9)	0.01582 (17)	
C(21)	0.73639 (12)	0.33649 (8)	0.28814 (11)	0.0232 (2)	
C(3)	0.11712 (11)	0.07211 (6)	0.37966 (10)	0.0189 (2)	
C(6)	−0.09818 (11)	0.17167 (7)	0.53542 (11)	0.0218 (2)	
C(5)	−0.05552 (11)	0.11665 (7)	0.46091 (10)	0.0204 (2)	
C(7)	−0.01276 (11)	0.24398 (7)	0.60500 (10)	0.0193 (2)	
C(14)	0.63655 (10)	0.52346 (6)	0.76214 (9)	0.01640 (18)	
C(2)	0.23832 (11)	0.08490 (6)	0.37008 (10)	0.0186 (2)	
H(14)	0.6955	0.2786	0.2865	0.031*	
H(15)	0.6802	0.3620	0.1945	0.031*	
H(16)	0.8329	0.3246	0.2981	0.030*	
H(13)	0.8799	0.4859	0.3958	0.022*	
H(12)	0.8801	0.5794	0.5735	0.021*	
H(11)	0.7731	0.6110	0.7334	0.019*	
H(10)	0.6273	0.5617	0.8363	0.020*	
H(9)	0.5028	0.4223	0.7652	0.019*	
H(7)	0.3521	0.3307	0.6380	0.017*	
H(23)	0.2722	0.0414	0.3209	0.024*	
H(24)	0.0524	0.0214	0.3345	0.023*	
H(27)	−0.1126	0.0658	0.4138	0.026*	
H(28)	−0.1878	0.1601	0.5383	0.027*	
H(5)	−0.0421	0.2858	0.6598	0.025*	
H(6)	0.1698	0.3124	0.6486	0.020*	
H(8)	0.5037	0.2828	0.4982	0.019*	

### Atomic displacement parameters (Å^2^)

**Table:**

	*U*^11^	*U*^22^	*U*^33^	*U*^12^	*U*^13^	*U*^23^
O(1)	0.0251 (3)	0.0216 (3)	0.0238 (3)	−0.0035 (2)	0.0176 (3)	−0.0051 (2)
N(1)	0.0156 (3)	0.0135 (3)	0.0136 (3)	−0.0013 (2)	0.0082 (2)	−0.0003 (2)
N(2)	0.0144 (3)	0.0174 (3)	0.0131 (3)	0.0008 (2)	0.0066 (2)	0.0003 (2)
C(20)	0.0124 (3)	0.0146 (3)	0.0115 (3)	0.0011 (2)	0.0051 (3)	0.0015 (2)
C(13)	0.0140 (3)	0.0175 (4)	0.0134 (3)	−0.0002 (3)	0.0065 (3)	−0.0007 (3)
C(11)	0.0154 (3)	0.0137 (3)	0.0127 (3)	−0.0002 (2)	0.0072 (3)	0.0008 (2)
C(9)	0.0160 (3)	0.0140 (3)	0.0111 (3)	−0.0016 (3)	0.0064 (3)	0.0014 (2)
C(12)	0.0127 (3)	0.0133 (3)	0.0123 (3)	0.0000 (2)	0.0053 (3)	0.0006 (2)
C(10)	0.0163 (3)	0.0131 (3)	0.0121 (3)	−0.0018 (2)	0.0078 (3)	−0.0001 (2)
C(19)	0.0144 (3)	0.0226 (4)	0.0134 (4)	0.0011 (3)	0.0066 (3)	0.0017 (3)
C(16)	0.0129 (3)	0.0158 (4)	0.0124 (3)	−0.0006 (2)	0.0043 (3)	0.0017 (2)
C(17)	0.0145 (3)	0.0197 (4)	0.0158 (4)	−0.0024 (3)	0.0058 (3)	0.0033 (3)
C(1)	0.0211 (4)	0.0154 (4)	0.0130 (3)	−0.0009 (3)	0.0094 (3)	−0.0003 (3)
C(18)	0.0150 (4)	0.0248 (4)	0.0153 (4)	−0.0012 (3)	0.0074 (3)	0.0044 (3)
C(15)	0.0165 (4)	0.0151 (4)	0.0142 (4)	−0.0013 (3)	0.0046 (3)	−0.0001 (3)
C(4)	0.0190 (4)	0.0164 (4)	0.0122 (3)	−0.0035 (3)	0.0070 (3)	0.0002 (3)
C(8)	0.0176 (4)	0.0160 (4)	0.0152 (4)	−0.0017 (3)	0.0086 (3)	−0.0001 (3)
C(21)	0.0245 (4)	0.0310 (5)	0.0188 (4)	−0.0020 (4)	0.0138 (3)	−0.0039 (3)
C(3)	0.0254 (4)	0.0166 (4)	0.0142 (4)	−0.0057 (3)	0.0086 (3)	−0.0022 (3)
C(6)	0.0182 (4)	0.0265 (5)	0.0227 (4)	−0.0041 (3)	0.0111 (3)	0.0017 (3)
C(5)	0.0201 (4)	0.0215 (4)	0.0192 (4)	−0.0070 (3)	0.0085 (3)	0.0001 (3)
C(7)	0.0197 (4)	0.0217 (4)	0.0196 (4)	0.0001 (3)	0.0115 (3)	0.0016 (3)
C(14)	0.0163 (4)	0.0174 (4)	0.0141 (4)	0.0003 (3)	0.0057 (3)	−0.0015 (3)
C(2)	0.0269 (4)	0.0164 (4)	0.0148 (4)	−0.0036 (3)	0.0115 (3)	−0.0036 (3)

### Geometric parameters (Å, °)

**Table:**

O(1)—C(1)	1.2629 (15)	C(17)—H(12)	1.009
N(1)—C(11)	1.3272 (14)	C(1)—C(2)	1.4549 (12)
N(1)—C(12)	1.4056 (11)	C(18)—H(13)	0.988
N(1)—H(8)	0.951	C(15)—C(14)	1.3743 (17)
N(2)—C(20)	1.3653 (13)	C(15)—H(11)	1.015
N(2)—C(19)	1.3187 (16)	C(4)—C(3)	1.4397 (16)
C(20)—C(12)	1.4240 (16)	C(4)—C(5)	1.4088 (17)
C(20)—C(16)	1.4174 (12)	C(8)—C(7)	1.3846 (17)
C(13)—C(12)	1.3797 (14)	C(8)—H(6)	0.991
C(13)—C(14)	1.4107 (12)	C(21)—H(14)	0.971
C(13)—H(9)	0.997	C(21)—H(15)	1.002
C(11)—C(10)	1.3955 (12)	C(21)—H(16)	0.997
C(11)—H(7)	0.982	C(3)—C(2)	1.3534 (18)
C(9)—C(10)	1.4584 (15)	C(3)—H(24)	0.997
C(9)—C(4)	1.4165 (12)	C(6)—C(5)	1.3791 (18)
C(9)—C(8)	1.4139 (15)	C(6)—C(7)	1.4016 (13)
C(10)—C(1)	1.4479 (15)	C(6)—H(28)	0.983
C(19)—C(18)	1.4238 (14)	C(5)—H(27)	0.969
C(19)—C(21)	1.5008 (17)	C(7)—H(5)	1.014
C(16)—C(17)	1.4196 (17)	C(14)—H(10)	1.038
C(16)—C(15)	1.4149 (15)	C(2)—H(23)	1.015
C(17)—C(18)	1.3653 (16)		
			
O(1)···C(13)^i^	3.4444 (14)	H(15)···C(6)^xii^	3.552
O(1)···C(11)^i^	3.4375 (13)	H(15)···H(10)^iv^	3.333
O(1)···H(15)^ii^	3.084	H(15)···H(23)^vii^	2.771
O(1)···H(10)^iii^	3.578	H(15)···H(28)^xii^	2.687
O(1)···H(9)^i^	2.505	H(15)···H(8)^i^	3.042
O(1)···H(7)^i^	2.701	H(16)···C(8)^v^	3.413
O(1)···H(6)^i^	2.964	H(16)···C(6)^v^	3.303
N(1)···C(16)^iv^	3.5486 (11)	H(16)···C(6)^xii^	3.285
N(1)···C(15)^iv^	3.4002 (10)	H(16)···C(5)^v^	3.536
N(1)···C(21)^ii^	3.5316 (12)	H(16)···C(7)^v^	3.240
N(1)···H(14)^ii^	2.834	H(16)···C(7)^xii^	3.381
N(1)···H(15)^ii^	3.289	H(16)···H(12)^viii^	3.078
N(1)···H(11)^iv^	3.353	H(16)···H(23)^vii^	3.507
N(2)···C(14)^iv^	3.3939 (10)	H(16)···H(28)^xii^	2.770
N(2)···H(10)^iv^	3.454	H(16)···H(5)^xii^	2.947
N(2)···H(28)^v^	3.450	H(13)···C(17)^viii^	2.949
N(2)···H(5)^v^	3.177	H(13)···C(18)^viii^	2.914
C(20)···C(13)^iv^	3.5462 (11)	H(13)···C(3)^vii^	3.307
C(20)···C(14)^iv^	3.5818 (10)	H(13)···C(2)^vii^	2.999
C(20)···H(27)^vi^	3.543	H(13)···H(13)^viii^	2.573
C(20)···H(5)^v^	3.523	H(13)···H(12)^viii^	2.609
C(13)···O(1)^ii^	3.4444 (14)	H(13)···H(23)^vii^	2.339
C(13)···C(20)^iv^	3.5462 (11)	H(13)···H(24)^vii^	2.977
C(13)···H(14)^ii^	3.574	H(13)···H(6)^iv^	3.088
C(13)···H(27)^vi^	3.135	H(12)···C(11)^iv^	3.116
C(11)···O(1)^ii^	3.4375 (13)	H(12)···C(9)^iv^	3.379
C(11)···C(17)^iv^	3.3368 (12)	H(12)···C(10)^iv^	3.413
C(11)···H(14)^ii^	3.305	H(12)···C(18)^viii^	3.339
C(11)···H(12)^iv^	3.116	H(12)···C(8)^iv^	3.081
C(11)···H(11)^iv^	3.371	H(12)···H(16)^viii^	3.078
C(9)···C(15)^iii^	3.5225 (12)	H(12)···H(13)^viii^	2.609
C(9)···H(12)^iv^	3.379	H(12)···H(7)^iv^	2.847
C(9)···H(11)^iii^	2.822	H(12)···H(24)^vi^	3.016
C(9)···H(10)^iii^	3.012	H(12)···H(6)^iv^	2.778
C(9)···H(5)^i^	3.563	H(11)···N(1)^iv^	3.353
C(12)···C(16)^iv^	3.4161 (10)	H(11)···C(11)^iv^	3.371
C(12)···C(15)^iv^	3.5772 (10)	H(11)···C(9)^ix^	2.822
C(12)···H(14)^ii^	3.111	H(11)···C(10)^ix^	3.559
C(12)···H(27)^vi^	3.523	H(11)···C(10)^iv^	3.538
C(10)···H(12)^iv^	3.413	H(11)···C(4)^ix^	3.074
C(10)···H(11)^iii^	3.559	H(11)···C(8)^ix^	2.825
C(10)···H(11)^iv^	3.538	H(11)···C(6)^ix^	3.351
C(10)···H(10)^iii^	2.823	H(11)···C(5)^ix^	3.333
C(19)···C(7)^v^	3.4127 (13)	H(11)···C(7)^ix^	3.089
C(19)···H(23)^vii^	3.279	H(11)···H(24)^vi^	3.319
C(19)···H(5)^v^	3.173	H(11)···H(27)^vi^	3.211
C(16)···N(1)^iv^	3.5486 (11)	H(11)···H(6)^ix^	3.248
C(16)···C(12)^iv^	3.4161 (10)	H(11)···H(8)^iv^	3.297
C(16)···H(7)^iv^	3.494	H(10)···O(1)^ix^	3.578
C(16)···H(24)^vi^	3.192	H(10)···N(2)^iv^	3.454
C(16)···H(27)^vi^	3.187	H(10)···C(9)^ix^	3.012
C(17)···C(11)^iv^	3.3368 (12)	H(10)···C(10)^ix^	2.823
C(17)···H(13)^viii^	2.949	H(10)···C(1)^ix^	2.827
C(17)···H(7)^iv^	2.957	H(10)···C(4)^ix^	3.154
C(17)···H(24)^vi^	3.108	H(10)···C(3)^ix^	3.069
C(17)···H(6)^iv^	3.268	H(10)···C(2)^ix^	2.893
C(1)···C(8)^i^	3.5076 (11)	H(10)···H(15)^iv^	3.333
C(1)···H(15)^ii^	3.564	H(10)···H(23)^ix^	3.428
C(1)···H(10)^iii^	2.827	H(10)···H(27)^vi^	3.149
C(1)···H(9)^i^	3.382	H(9)···O(1)^ii^	2.505
C(1)···H(7)^i^	3.336	H(9)···C(1)^ii^	3.382
C(1)···H(6)^i^	2.791	H(9)···C(18)^iv^	3.537
C(18)···C(18)^viii^	3.5100 (14)	H(9)···C(2)^ii^	3.492
C(18)···H(13)^viii^	2.914	H(9)···H(23)^ii^	2.837
C(18)···H(12)^viii^	3.339	H(9)···H(28)^vi^	3.517
C(18)···H(9)^iv^	3.537	H(7)···O(1)^ii^	2.701
C(18)···H(7)^iv^	3.410	H(7)···C(16)^iv^	3.494
C(18)···H(23)^vii^	2.800	H(7)···C(17)^iv^	2.957
C(18)···H(5)^v^	3.532	H(7)···C(1)^ii^	3.336
C(18)···H(6)^iv^	3.424	H(7)···C(18)^iv^	3.410
C(15)···N(1)^iv^	3.4002 (10)	H(7)···C(2)^ii^	3.520
C(15)···C(9)^ix^	3.5225 (12)	H(7)···H(12)^iv^	2.847
C(15)···C(12)^iv^	3.5772 (10)	H(7)···H(23)^ii^	3.168
C(15)···C(4)^ix^	3.5751 (11)	H(23)···C(19)^xiii^	3.279
C(15)···H(24)^vi^	3.333	H(23)···C(18)^xiii^	2.800
C(15)···H(27)^vi^	2.802	H(23)···C(21)^xiii^	3.299
C(15)···H(8)^iv^	3.466	H(23)···H(15)^xiii^	2.771
C(4)···C(15)^iii^	3.5751 (11)	H(23)···H(16)^xiii^	3.507
C(4)···H(11)^iii^	3.074	H(23)···H(13)^xiii^	2.339
C(4)···H(10)^iii^	3.154	H(23)···H(10)^iii^	3.428
C(4)···H(27)^x^	3.257	H(23)···H(9)^i^	2.837
C(4)···H(5)^i^	3.144	H(23)···H(7)^i^	3.168
C(8)···C(1)^ii^	3.5076 (11)	H(23)···H(6)^i^	2.784
C(8)···C(2)^ii^	3.5355 (13)	H(24)···C(16)^xv^	3.192
C(8)···H(16)^xi^	3.413	H(24)···C(17)^xv^	3.108
C(8)···H(12)^iv^	3.081	H(24)···C(15)^xv^	3.333
C(8)···H(11)^iii^	2.825	H(24)···C(6)^x^	3.180
C(21)···N(1)^i^	3.5316 (12)	H(24)···C(5)^x^	3.052
C(21)···C(6)^v^	3.5060 (14)	H(24)···H(13)^xiii^	2.977
C(21)···C(7)^v^	3.5654 (13)	H(24)···H(12)^xv^	3.016
C(21)···H(23)^vii^	3.299	H(24)···H(11)^xv^	3.319
C(21)···H(28)^xii^	3.194	H(24)···H(27)^x^	2.858
C(21)···H(8)^i^	3.510	H(24)···H(28)^x^	3.111
C(3)···C(5)^x^	3.5552 (15)	H(24)···H(5)^i^	3.375
C(3)···H(13)^xiii^	3.307	H(27)···C(20)^xv^	3.543
C(3)···H(10)^iii^	3.069	H(27)···C(13)^xv^	3.135
C(3)···H(27)^x^	3.094	H(27)···C(12)^xv^	3.523
C(3)···H(5)^i^	3.092	H(27)···C(16)^xv^	3.187
C(3)···H(6)^i^	3.329	H(27)···C(15)^xv^	2.802
C(6)···C(21)^xi^	3.5060 (14)	H(27)···C(4)^x^	3.257
C(6)···H(14)^xi^	3.070	H(27)···C(3)^x^	3.094
C(6)···H(15)^xiv^	3.552	H(27)···C(5)^x^	3.226
C(6)···H(16)^xi^	3.303	H(27)···C(14)^xv^	2.765
C(6)···H(16)^xiv^	3.285	H(27)···H(11)^xv^	3.211
C(6)···H(11)^iii^	3.351	H(27)···H(10)^xv^	3.149
C(6)···H(24)^x^	3.180	H(27)···H(24)^x^	2.858
C(5)···C(3)^x^	3.5552 (15)	H(27)···H(27)^x^	3.019
C(5)···H(14)^xi^	3.467	H(28)···N(2)^xi^	3.450
C(5)···H(16)^xi^	3.536	H(28)···C(21)^xiv^	3.194
C(5)···H(11)^iii^	3.333	H(28)···H(14)^xi^	3.042
C(5)···H(24)^x^	3.052	H(28)···H(14)^xiv^	3.593
C(5)···H(27)^x^	3.226	H(28)···H(15)^xiv^	2.687
C(7)···C(19)^xi^	3.4127 (13)	H(28)···H(16)^xiv^	2.770
C(7)···C(21)^xi^	3.5654 (13)	H(28)···H(9)^xv^	3.517
C(7)···H(14)^xi^	3.499	H(28)···H(24)^x^	3.111
C(7)···H(16)^xi^	3.240	H(5)···N(2)^xi^	3.177
C(7)···H(16)^xiv^	3.381	H(5)···C(20)^xi^	3.523
C(7)···H(11)^iii^	3.089	H(5)···C(9)^ii^	3.563
C(14)···N(2)^iv^	3.3939 (10)	H(5)···C(19)^xi^	3.173
C(14)···C(20)^iv^	3.5818 (10)	H(5)···C(18)^xi^	3.532
C(14)···H(27)^vi^	2.765	H(5)···C(4)^ii^	3.144
C(2)···C(8)^i^	3.5355 (13)	H(5)···C(3)^ii^	3.092
C(2)···H(13)^xiii^	2.999	H(5)···C(2)^ii^	3.427
C(2)···H(10)^iii^	2.893	H(5)···H(16)^xiv^	2.947
C(2)···H(9)^i^	3.492	H(5)···H(24)^ii^	3.375
C(2)···H(7)^i^	3.520	H(6)···O(1)^ii^	2.964
C(2)···H(5)^i^	3.427	H(6)···C(17)^iv^	3.268
C(2)···H(6)^i^	2.689	H(6)···C(1)^ii^	2.791
H(14)···N(1)^i^	2.834	H(6)···C(18)^iv^	3.424
H(14)···C(13)^i^	3.574	H(6)···C(3)^ii^	3.329
H(14)···C(11)^i^	3.305	H(6)···C(2)^ii^	2.689
H(14)···C(12)^i^	3.111	H(6)···H(13)^iv^	3.088
H(14)···C(6)^v^	3.070	H(6)···H(12)^iv^	2.778
H(14)···C(5)^v^	3.467	H(6)···H(11)^iii^	3.248
H(14)···C(7)^v^	3.499	H(6)···H(23)^ii^	2.784
H(14)···H(28)^v^	3.042	H(8)···C(15)^iv^	3.466
H(14)···H(28)^xii^	3.593	H(8)···C(21)^ii^	3.510
H(14)···H(8)^i^	3.027	H(8)···H(14)^ii^	3.027
H(15)···O(1)^i^	3.084	H(8)···H(15)^ii^	3.042
H(15)···N(1)^i^	3.289	H(8)···H(11)^iv^	3.297
H(15)···C(1)^i^	3.564		
			
C(11)—N(1)—C(12)	124.89 (9)	C(19)—C(18)—H(13)	118.0
C(11)—N(1)—H(8)	115.8	C(17)—C(18)—H(13)	121.9
C(12)—N(1)—H(8)	118.6	C(16)—C(15)—C(14)	119.59 (8)
C(20)—N(2)—C(19)	118.26 (8)	C(16)—C(15)—H(11)	119.1
N(2)—C(20)—C(12)	117.74 (8)	C(14)—C(15)—H(11)	121.3
N(2)—C(20)—C(16)	123.55 (10)	C(9)—C(4)—C(3)	119.20 (10)
C(12)—C(20)—C(16)	118.70 (9)	C(9)—C(4)—C(5)	120.42 (10)
C(12)—C(13)—C(14)	119.82 (10)	C(3)—C(4)—C(5)	120.37 (8)
C(12)—C(13)—H(9)	120.2	C(9)—C(8)—C(7)	121.54 (8)
C(14)—C(13)—H(9)	120.0	C(9)—C(8)—H(6)	119.9
N(1)—C(11)—C(10)	124.03 (10)	C(7)—C(8)—H(6)	118.6
N(1)—C(11)—H(7)	115.9	C(19)—C(21)—H(14)	111.1
C(10)—C(11)—H(7)	120.0	C(19)—C(21)—H(15)	114.3
C(10)—C(9)—C(4)	119.22 (9)	C(19)—C(21)—H(16)	112.8
C(10)—C(9)—C(8)	123.56 (7)	H(14)—C(21)—H(15)	105.9
C(4)—C(9)—C(8)	117.22 (9)	H(14)—C(21)—H(16)	105.7
N(1)—C(12)—C(20)	115.74 (9)	H(15)—C(21)—H(16)	106.5
N(1)—C(12)—C(13)	123.75 (10)	C(4)—C(3)—C(2)	122.09 (8)
C(20)—C(12)—C(13)	120.47 (8)	C(4)—C(3)—H(24)	115.5
C(11)—C(10)—C(9)	120.13 (9)	C(2)—C(3)—H(24)	122.4
C(11)—C(10)—C(1)	119.41 (10)	C(5)—C(6)—C(7)	119.01 (11)
C(9)—C(10)—C(1)	120.45 (7)	C(5)—C(6)—H(28)	119.9
N(2)—C(19)—C(18)	122.17 (10)	C(7)—C(6)—H(28)	121.1
N(2)—C(19)—C(21)	117.77 (9)	C(4)—C(5)—C(6)	121.13 (9)
C(18)—C(19)—C(21)	120.05 (11)	C(4)—C(5)—H(27)	119.0
C(20)—C(16)—C(17)	116.63 (9)	C(6)—C(5)—H(27)	119.9
C(20)—C(16)—C(15)	120.07 (10)	C(8)—C(7)—C(6)	120.67 (11)
C(17)—C(16)—C(15)	123.29 (8)	C(8)—C(7)—H(5)	118.7
C(16)—C(17)—C(18)	119.26 (8)	C(6)—C(7)—H(5)	120.6
C(16)—C(17)—H(12)	119.7	C(13)—C(14)—C(15)	121.26 (9)
C(18)—C(17)—H(12)	121.0	C(13)—C(14)—H(10)	119.1
O(1)—C(1)—C(10)	123.19 (8)	C(15)—C(14)—H(10)	119.6
O(1)—C(1)—C(2)	119.75 (10)	C(1)—C(2)—C(3)	121.72 (10)
C(10)—C(1)—C(2)	117.07 (10)	C(1)—C(2)—H(23)	116.2
C(19)—C(18)—C(17)	120.10 (11)	C(3)—C(2)—H(23)	122.1
			
C(11)—N(1)—C(12)—C(20)	−161.97 (7)	C(8)—C(9)—C(10)—C(1)	−178.44 (7)
C(11)—N(1)—C(12)—C(13)	15.91 (12)	C(4)—C(9)—C(8)—C(7)	0.50 (12)
C(12)—N(1)—C(11)—C(10)	173.84 (7)	C(8)—C(9)—C(4)—C(3)	−178.14 (7)
C(20)—N(2)—C(19)—C(18)	1.52 (12)	C(8)—C(9)—C(4)—C(5)	0.52 (12)
C(20)—N(2)—C(19)—C(21)	−177.56 (7)	C(11)—C(10)—C(1)—O(1)	−3.56 (12)
C(19)—N(2)—C(20)—C(12)	178.75 (7)	C(11)—C(10)—C(1)—C(2)	176.36 (7)
C(19)—N(2)—C(20)—C(16)	−0.26 (11)	C(9)—C(10)—C(1)—O(1)	175.20 (8)
N(2)—C(20)—C(12)—N(1)	−2.91 (10)	C(9)—C(10)—C(1)—C(2)	−4.88 (11)
N(2)—C(20)—C(12)—C(13)	179.13 (7)	N(2)—C(19)—C(18)—C(17)	−1.38 (13)
N(2)—C(20)—C(16)—C(17)	−1.12 (11)	C(21)—C(19)—C(18)—C(17)	177.69 (8)
N(2)—C(20)—C(16)—C(15)	178.12 (7)	C(20)—C(16)—C(17)—C(18)	1.24 (11)
C(12)—C(20)—C(16)—C(17)	179.88 (7)	C(20)—C(16)—C(15)—C(14)	1.93 (11)
C(12)—C(20)—C(16)—C(15)	−0.89 (11)	C(17)—C(16)—C(15)—C(14)	−178.89 (7)
C(16)—C(20)—C(12)—N(1)	176.15 (6)	C(15)—C(16)—C(17)—C(18)	−177.97 (7)
C(16)—C(20)—C(12)—C(13)	−1.80 (11)	C(16)—C(17)—C(18)—C(19)	−0.10 (12)
C(12)—C(13)—C(14)—C(15)	−2.37 (11)	O(1)—C(1)—C(2)—C(3)	−174.35 (8)
C(14)—C(13)—C(12)—N(1)	−174.36 (7)	C(10)—C(1)—C(2)—C(3)	5.73 (12)
C(14)—C(13)—C(12)—C(20)	3.42 (11)	C(16)—C(15)—C(14)—C(13)	−0.32 (12)
N(1)—C(11)—C(10)—C(9)	−173.35 (7)	C(9)—C(4)—C(3)—C(2)	−1.72 (13)
N(1)—C(11)—C(10)—C(1)	5.42 (12)	C(9)—C(4)—C(5)—C(6)	−0.81 (13)
C(10)—C(9)—C(4)—C(3)	2.44 (12)	C(3)—C(4)—C(5)—C(6)	177.83 (8)
C(10)—C(9)—C(4)—C(5)	−178.90 (8)	C(5)—C(4)—C(3)—C(2)	179.62 (8)
C(4)—C(9)—C(10)—C(11)	179.70 (7)	C(9)—C(8)—C(7)—C(6)	−1.25 (13)
C(4)—C(9)—C(10)—C(1)	0.94 (12)	C(4)—C(3)—C(2)—C(1)	−2.51 (13)
C(10)—C(9)—C(8)—C(7)	179.89 (8)	C(5)—C(6)—C(7)—C(8)	0.94 (14)
C(8)—C(9)—C(10)—C(11)	0.32 (12)	C(7)—C(6)—C(5)—C(4)	0.08 (14)

Symmetry codes: (i) *x*, −*y*+1/2, *z*−1/2; (ii) *x*, −*y*+1/2, *z*+1/2; (iii) −*x*+1, *y*−1/2, −*z*+3/2; (iv) −*x*+1, −*y*+1, −*z*+1; (v) *x*+1, *y*, *z*; (vi) *x*+1, −*y*+1/2, *z*+1/2; (vii) −*x*+1, *y*+1/2, −*z*+1/2; (viii) −*x*+2, −*y*+1, −*z*+1; (ix) −*x*+1, *y*+1/2, −*z*+3/2; (x) −*x*, −*y*, −*z*+1; (xi) *x*−1, *y*, *z*; (xii) *x*+1, −*y*+1/2, *z*−1/2; (xiii) −*x*+1, *y*−1/2, −*z*+1/2; (xiv) *x*−1, −*y*+1/2, *z*+1/2; (xv) *x*−1, −*y*+1/2, *z*−1/2.

### Hydrogen-bond geometry (Å, °)

**Table:**

*D*—H···*A*	*D*—H	H···*A*	*D*···*A*	*D*—H···*A*
N1—H8···O1	0.951	1.863	2.6094 (11)	133.3
N1—H8···N2	0.951	2.278	2.6714 (14)	103.9
